# Direct comparison of MRI and X-ray CT technologies for 3D imaging of root systems in soil: potential and challenges for root trait quantification

**DOI:** 10.1186/s13007-015-0060-z

**Published:** 2015-03-11

**Authors:** Ralf Metzner, Anja Eggert, Dagmar van Dusschoten, Daniel Pflugfelder, Stefan Gerth, Ulrich Schurr, Norman Uhlmann, Siegfried Jahnke

**Affiliations:** Institute of Bio- and Geosciences, IBG-2: Plant Sciences, Forschungszentrum Jülich GmbH, Wilhelm-Jonen- Str., 52425 Jülich, Germany; Development Center X-Ray Technology EZRT, Fraunhofer Institute for Integrated Systems IIS, Flugplatzstraße 75, 90768 Fürth, Germany

**Keywords:** X-ray Computed Tomography (CT), Magnetic Resonance Imaging (MRI), Root system architecture, Common bean (*Phaseolus vulgaris* L.) 3D imaging, Roots in soil, Non-destructive

## Abstract

**Background:**

Roots are vital to plants for soil exploration and uptake of water and nutrients. Root performance is critical for growth and yield of plants, in particular when resources are limited. Since roots develop in strong interaction with the soil matrix, tools are required that can visualize and quantify root growth in opaque soil at best in 3D. Two modalities that are suited for such investigations are X-ray Computed Tomography (CT) and Magnetic Resonance Imaging (MRI). Due to the different physical principles they are based on, these modalities have their specific potentials and challenges for root phenotyping. We compared the two methods by imaging the same root systems grown in 3 different pot sizes with inner diameters of 34 mm, 56 mm or 81 mm.

**Results:**

Both methods successfully visualized roots of two weeks old bean plants in all three pot sizes. Similar root images and almost the same root length were obtained for roots grown in the small pot, while more root details showed up in the CT images compared to MRI. For the medium sized pot, MRI showed more roots and higher root lengths whereas at some spots thin roots were only found by CT and the high water content apparently affected CT more than MRI. For the large pot, MRI detected much more roots including some laterals than CT.

**Conclusions:**

Both techniques performed equally well for pots with small diameters which are best suited to monitor root development of seedlings. To investigate specific root details or finely graduated root diameters of thin roots, CT was advantageous as it provided the higher spatial resolution. For larger pot diameters, MRI delivered higher fractions of the root systems than CT, most likely because of the strong root-to-soil contrast achievable by MRI. Since complementary information can be gathered with CT and MRI, a combination of the two modalities could open a whole range of additional possibilities like analysis of root system traits in different soil structures or under varying soil moisture.

**Electronic supplementary material:**

The online version of this article (doi:10.1186/s13007-015-0060-z) contains supplementary material, which is available to authorized users.

## Background

Roots are vital for higher plants to gather water and nutrients and thus critical for performance and productivity [1]. Considering the impact on crop yields, more knowledge is needed about root system development belowground and interaction with the surrounding soil [2-4]. Approaches to investigate roots and their development reach from excavation and measurement of root system traits with manual or optical methods [5-7], mini-rhizotrons [7] or trenches dug into the ground [1], transparent artificial growth media [8,9] to soil based 2D growth monitoring in rhizoboxes [1,10,11]. All these are valid for answering specific questions concerning root development; however, none of these is able to follow 3D root development in soil where a multitude of biotic and abiotic interaction takes place. Quantitative knowledge of 3D root traits would help to achieve better mechanistic understanding of root architecture as it develops in a soil environment. Non-invasive approaches which have been used already to investigate plant roots in soil are magnetic resonance imaging (MRI) [12-15], neutron computed tomography [16,17] and X-ray computed tomography (CT) [3,18-21]. Neutron tomography requires access to a nuclear reactor or a high energy particle accelerator whereas MRI and CT on the other hand, though still expensive, are becoming available to a steadily growing number of plant biologists.

CT employs an X-ray beam passing through the sample which absorbs part of it thereby reducing the intensity of the beam. This process is called attenuation. The sample is rotated between an X-ray source and a detector, recording series of 2D projections of the object from which a 3D volume dataset can be reconstructed. X-ray attenuation is mainly determined by material properties, in particular electron-density. Thereby the internal structure of the sample becomes visible by contrast according to density and atomic number of the elements [22]. Further information about the basics of CT can be found in various articles [23,24] or text books [25]. Recent applications in plant sciences include lateral root development [26] or root elongation rates [27]. A number of other applications covering a range of plant species and root traits are reviewed in [3]. A major problem is the often very similar attenuation of roots and some structures in the soil such as water filled pores [3]. This leads to low contrast hindering simple and straightforward segmentation of the roots from the soil background. Different approaches were tried to tackle this problem, such as adjusting soil humidity to optimize contrast [28] or sophisticated software tools for data analysis [29]. Together with scanners that can produce high resolution scans in short time [3] these approaches may lead to a wider use of CT in plant sciences.

MRI is based on the magnetic moment of atomic nuclei like ^1^H (protons) which are highly abundant in living tissues particularly in water molecules. The magnetic moment can be manipulated using strong magnetic fields and radio frequency fields to produce 3D datasets of samples. The magnetic fields require a substrate with low ferro-magnetic particle content for high quality images. A range of contrast parameters can be exploited, highlighting differences within the sample such as density of the protons or their physical and chemical micro-environment. This can be exploited to produce a strong difference between ‘root water signal’ and ‘soil water signal’ which provides a very high contrast between roots and soil background [13,14]. The basic principles of MRI and its use in biomedical sciences are described in detail in several textbooks [30,31] or review articles of plant biology [12,32-34]. Research applications to plant roots range from phytopathology [35], across storage root internal structures [15] to combined studies with positron emission tomography for structure function relations [13]. Also water mobility in roots and soil has been shown to be detectable with MRI [36,37].

Both CT and MRI are able to image root systems in soil. Their fundamental different physical principles give each of them a unique perspective but also different challenges for application in the plant sciences. Therefore, a direct comparison of both techniques using the same samples and analyzing for the same root system may help to decide which method is better suited for a specific research question. A whole range of instrument parameters can affect root imaging with either method but also sample parameters such as soil type [14,38] or moisture [28]. Pot sizes, on the other hand has rarely been considered despite its importance for root system and plant development [39] making it an important parameter of experimental design. Since pot size may also affect root imaging and data analysis depending on the applied method, we focus in our CT-MRI comparison approach on this parameter using common bean (*Phaseolus vulgaris*) as a model plant. We evaluated both techniques for imaging roots in soil, segmentation of roots from the soil background and extracting root length as a basic parameter of root system architecture (RSA). For comparison the roots were finally excavated, washed and scanned using WinRHIZO as a widely used standard technique.

## Results and discussion

We compared reconstructed root systems of bean plants in pots of three different sizes imaged by both CT and MRI. The small pots had an inner diameter (I.D.) of 34 mm and a height of 200 mm which could be used to study early growth stage in crop plants. Pots of this size or smaller were also used in other high resolution CT studies [3,19]. The medium pots with an I.D. of 56 mm and a height of 200 mm would allow plant growth for a few weeks or even a whole growth cycle of small plants. The large pots had I.D. of 81 mm and were 300 mm high, thereby suited to cultivate plants for a longer time and even plants with large storage roots like sugar beet [15]. Here, we focused on the potentials and challenges for image quality and extracting root length as a major trait of RSA [1,11] to compare the two imaging modalities.

### CT imaging of roots is based on high spatial resolution and a good segmentation procedure

**Figure 1:**
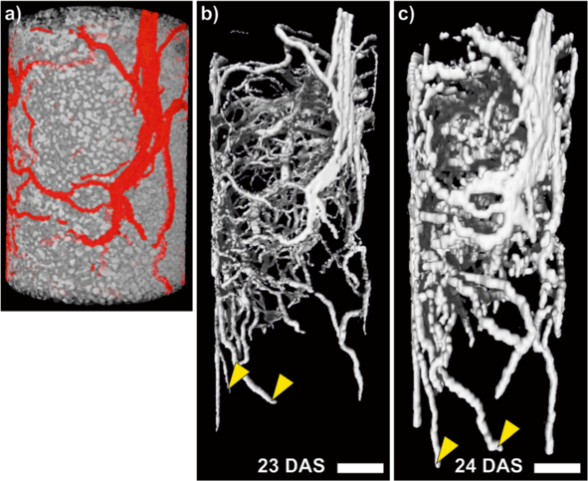
**CT and MRI images of soil and bean roots in a small pot.** The same plant grown in a soil filled pot with an inner diameter (I.D.) of 34 mm and a height of 200 mm was imaged sequentially with X-ray Computed Tomography (CT) and Magnetic Resonance Imaging (MRI). **(a)** Shows the upper part of the soil column below the pot material imaged with CT (gray-scale) measured with voxel size of 28 × 28 × 28 μm^3^ with the roots of a bean plant (*Phaseolus vulgaris* L.) highlighted (red). **(b)** The root system segmented from the same CT image as in **(a)** on a voxel size of 56 × 56 × 56 μm^3^. **(c)** Shows the same root system imaged one day later with MRI and a voxel size of 333 × 333 × 1000 μm^3^ with the last value representing the vertical dimension. Arrowheads denote the same roots in both images. DAS: days after sowing Scale bar: 10 mm.

An example of a CT image with a spatial resolution of 28 μm (voxel size) is shown in Figure 1a which was obtained from a bean plant growing in a small pot. Roots that grew at the inner surface of the pot can be directly recognized by removing the pot material from the image without special segmentation efforts (Figure 1a). Due to their elongated shape they can be distinguished visually from sand grains and darker areas where air or water filled pores and finer soil particles were located. The ability to image both soil structures and plant roots is a very intriguing feature of CT [3,40,41]. However, roots developing as a 3D structure in a soil filled pot have to be detected by signal contrast based on X-ray attenuation which may be low between particular soil structures such as water filled pores and the roots [3,28]. Segmentation of the roots from the soil background is therefore a major step in extracting root structures and traits from CT images. So far many different approaches exist for this step in data analysis, exploiting different algorithms and filters in manual [19,40,42] or automated fashion [43,44]. However, a standard procedure remains yet to be defined. Figure 1b shows the result of a root segmentation procedure applied here to the dataset partly shown in Figure 1a. The segmentation follows an image analysis procedure including threshold filtering for manually selected grey values and for objects of a size and (cylindrical) shape typical for roots [45]. For the root segmentation procedure the spatial resolution of the images had to be reduced by a factor of 2 in each dimension because otherwise it would not have been possible to run the analysis on a standard workstation-sized computer with enhanced memory. The 3D rendered image of the segmented root system (Figure 1b) shows not only the roots at the soil surface but also a range of roots of variable diameter deep in the soil core. The 3D visualization allowed identification of primary, basal and hypocotyl-borne roots [46] by tracing them back to their respective origin (data not shown). In this way, the lowest root tips were identified as belonging to basal roots or the primary root (Figure 1b) while the thinner roots in the upper part of Figure 1b were lateral roots of each of these root types. The high spatial resolution of CT shown here also enables a precise mapping of root diameters.

The limit for the thinnest observable root is determined mainly by two factors: contrast (between roots and soil background) and spatial resolution (voxel size). Both factors influence the probability that a structure can be recognized as a root and thereby segmented. Indirectly, resolution may also influence contrast as voxels comprised of different materials with attenuation both higher and lower than roots (e.g. air and solid soil particles) may result in similar attenuation values as roots, the so called partial volume effect. Therefore a high spatial resolution may also enhance contrast. Other factors include signal-to-noise ratio and imaging artefacts such as beam hardening [3,41] but these were found to be of less importance in this study. The thinnest roots detected here were about 100 μm in diameter, corresponding to 2 voxel diameters in the segmentation. This relation between voxel resolution and minimal detectable object size fits well with the values found by other authors [41,47]. On the other hand, not all roots of 100–250 μm thick were quantitatively detected: when comparing the CT data with WinRHIZO data only about 70% of the total root length was found by CT segmentation (Table 1). However, for WinRHIZO roots classes thicker than 300 μm 78% and for those thicker than 400 μm 92% were found by CT. These values are in the order of the 86% Flavel et al. [19] found for wheat roots thicker than 250 μm in similarly sized pots considering the differences in plant species, scanner type and segmentation procedure. The imaging resolution used here was two times better than that achieved by Flavel et al. [19]; we therefore expect that, when the segmentation procedure can be further improved, a much larger fraction of a root system can be detected, at least in small pots. Other authors found values above 90% [48] but, according to their images, the segmentation between roots and water filled pores may have been suboptimal and lead to an overestimation of root length. To evaluate the effectiveness of the different segmentation approaches it might be useful to compare them directly on the same datasets as has been exemplarily attempted for soil parameter quantification with CT [49].

**Table 1:** **Root traits calculated from CT and MRI root images of the volumes shown in Figures** 1 **,** 2 **and** 3

	**Small pots (I.D. 34 mm)**			**Medium pots (I.D. 56)**			**Large pots (I.D. 81)**	
**Measurement modality**	**CT**	**MRI**	**Win-RHIZO**	**CT**	**MRI**	**Win-RHIZO**	**CT**	**MRI**
**Total root length [mm]**	2775	2910	4012	2787	4602	4855	6632	10496
**Percent root length of WinRHIZO [%]**	69	73		57	95			

### MRI imaging of roots is based on high root to soil contrast

The MRI image (Figure 1c) showed a similar 3D structure of the root system as segmented from the CT data (Figure 1b). Due to high contrast between roots and soil background, root segmentation from an MRI image is not necessary which means that Figure 1c shows the raw data with only a noise cut-off and no further processing. While the MRI scan took about as long as the CT scan, it must be noted that for the CT scan it took another two hours to reach the segmented image shown in Figure 1b. Though the root images from CT and MRI appear rather similar, there are some distinct differences due to fundamental differences in the imaging and segmentation principles. Roots which appear to be very thin in CT often look much thicker in MRI. This is caused by the much coarser spatial resolution of MRI (Figure 1c) with a voxel size of 330 × 330 × 1000 μm^3^, so any root detected is shown with voxels of this size whereas in the CT image (Figure 1b) the voxel size was 56 × 56 × 56 μm^3^. As already stated, in MRI the root detection is based on contrast against soil background which in this study was about two orders of magnitude, rather than on geometrical recognition as in CT. That means that roots even thinner than voxel resolution can be recognized as roots when only a (detectable) small fraction of the actual volume is comprised of root tissue. Therefore parts of the roots displayed in Figure 1c as one voxel thick were in reality thinner due to this sub-voxel resolution. We estimated that the thinnest roots detected with MRI were about 250 μm in diameter. The root length found with MRI was still only 70% of the total root length found with WinRHIZO after harvest (Table 1). Upon taking only the length of roots in the WinRHIZO classes thicker than 400 μm into account this fraction was 96%. Therefore, similar to CT, roots above this diameter appear to be largely detected, and in bean plants the primary, basal and hypocotyl borne roots are typically all above this threshold [46], the major root structure can be visualized in 3D. The high contrast between roots and soil background in MRI results from the fact that the signal decay from soil water is much faster than that from roots, so with the appropriate measurement settings only the latter is recorded. Since the signal decay of the soil water depends on soil properties, the measurement settings need to be adjusted adequately to the specific substrate. For MRI imaging of roots in soil the choice of substrate is important also because, due to the strong magnetic fields required for the measurements, disturbances of these fields have adverse effects on image quality. Ferromagnetic particles are thus most critical and their presence in the substrate for MRI should be minimized. This can either be done by selecting soils with naturally low content of ferromagnetic particles [14] or remove them from the sieved substrate with a simple arrangement of permanent magnets [15] as was used in this study. Paramagnetic ions such as Mn^2+^ described to be also problematic by some authors [50,51] did not appear to have any strong effect in our studies as demonstrated by the good image quality in the chosen soil (Figure 1c). The removal process for ferromagnetic particles led to a sieved and homogenized soil, and this substrate with good MRI properties was also used for CT. In contrast to pure MRI studies on roots [15,35] a higher fraction of coarse sand was used here, which may cause problems for plant cultivation because of low water retention capacity. However, pretests had shown that this allowed better segmentation results for CT due to higher contrast between roots and substrate, whereas MRI image quality was only marginally affected. Homogenized substrates appears to be advantageous for CT as it provides a rather homogenous background with small pores optimal for segmentation, which may be the reason why this type of substrate was also used in many CT studies targeting finer roots [28,52,53]. The need of homogenized substrates seems to be a common limitation for current MRI or CT applications in plant studies. Compared to natural soil from the field, which would be the ultimate condition to grow e.g. crop plants in, measure and characterize root traits, sieved soil comprises loss of texture and aggregate structure. Nevertheless, both methods are in principle able to investigate roots in soil cores collected in the field, as has been shown for CT [44] and also in a first MRI experiment in our lab (data not shown).

### For small pots both modalities recovered a comparable fraction of a root system with more details visible in the CT

When comparing the root images provided by both techniques, it is obvious that CT (Figure1b) with its higher spatial resolution showed many of the thinner roots with a higher level of detail than MRI (Figure 1c). For studies targeting different root classes in plants such as bean [46] or maize [54] this feature may be advantageous for identification and tracking of lateral roots of each class which are difficult to follow with MRI. Also thinner root systems in general may be investigated with CT as shown for *Arabidopsis* roots [55] which can be hardly achieved with MRI.

Visual comparison between root systems in 3D can be quite useful for qualitative description of treatment effects but, in addition to that, a quantitative evaluation of trait development is required. For the root system in the small pots shown in Figure 1, a root length of 2.8 m with CT and 2.9 m with MRI on the following day was found (Table 1) fitting well with the slightly longer roots in the lower part of the pot shown with MRI (Figure 1c) compared to CT (Figure 1b). Considering this, the fraction of the WinRHIZO-detected roots found by CT and MRI (69% and 73% respectively; Table 1) was almost identical, i.e. for such a (small) pot there was no obvious difference between MRI and CT in acquiring root lengths. Another experiment with a similar plant yielded longer total root length for all methods (Additional file 1), but the fraction of the roots found by WinRHIZO of CT (61%) and MRI (71%) was similar to that in Figure 1.

### For medium sized pots, CT showed more details but MRI revealed a larger fraction of the root system

**Figure 2:**
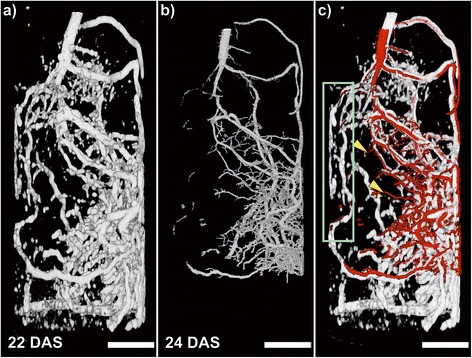
**MRI and CT images of a bean root system in a medium soil filled pot.** The same plant grown in a soil filled pot with an I.D. of 56 mm and a height of 200 mm was imaged sequentially by MRI and CT. **(a)** MRI image measured with a voxel size 375 × 375 × 1000 μm^3^. The CT image **(b)** shows the root system measured of the same plant segmented on a voxel size of 68 × 68 × 68 μm^3^. Roots in the lowest part of the pot could not be segmented and are therefore not shown. **(c)** CT-MRI co-registration: the CT image is in red and the MRI image in grey. Arrowheads highlight roots visible in CT but not in MRI. Box highlights area where few roots are visible in CT. Scale bar: 10 mm.

A bean plant growing in a medium sized pot (I.D. 56 mm, height 200 mm) was imaged with MRI (Figure 2a) and CT (Figure 2b). A larger pot size is needed for working with larger root systems and later developmental stages but also for bulky belowground structures such as storage organs to minimize growth limitations due to pot size [15,39]. However, the pot size affects also the quality of root imaging and segmentation of both CT and MRI. Either modality produced images of the root system of sufficient quality to see the overall structure of the specimen (Figure 2a,b). In contrast to the smaller pots, distinct differences can be seen in the overlay of the CT and MRI images (Figure 2c). The CT again showed detailed structures, a few of which were not visible in the MRI image such as small roots in the center. While it is possible that these have grown over the two days between the measurements, it is more likely that they were just too thin to be detected with MRI. Considering that the spatial resolution of MRI on the medium sized pots was only slightly lower than for the small ones, we estimated that roots above 280 μm should be largely detectable. Root segmentation from the CT dataset was more difficult for the medium pots compared to the small ones since resolution was limited to a voxel size of 68 μm due to the tradeoff between resolution and sample size apparent for a given CT system [3,24].

On the other hand, many roots detected with MRI also appeared to be successfully segmented with CT (Figure 2c) with two significant exceptions: on the left hand side and the bottom ~20 mm of the pot (Figure 2c) almost no roots could be successfully segmented from CT data, while MRI showed an abundance of roots. The lack of segmentation of roots from the CT image at these different positions can be taken as an example to explain different challenges frequently met when extracting roots from CT measurements. High water content can be an issue for segmentation as attenuation of roots and water-filled pores may overlap [28,42]. Gravity will inevitably lead to higher water content at the bottom, thereby degrading CT root segmentation particularly at the bottom of the pot; as dry soil also may increase artefacts from air filled pores in segmentation, a compromise may be to keep the soil in the pot at the soil-specific field capacity [28]. However, especially in higher pots needed to study the development of long roots or in sandy soils, the problem of water gradients in the pot still remains leading to segmentation problems towards the bottom of the pot. Additionally, water uptake by plants would also alter water distribution in a pot. It is therefore advisable to measure these effects for the chosen substrate-pot combination, water contents, CT-scanner and imaging system as a very basic control. Since the lowest part of a pot is the most likely to cause segmentation problems with CT, Figure 2b showed no roots in this area whereas a large number of roots was monitored at the bottom of the pot with MRI (Figure 2a). The MRI image also showed more unconnected voxels in this area with grey values similar to the roots compared to the upper part. We observed such unconnected voxels to be an artefact in MRI in the soil mixture used here when the water content surpassed about 10% to 15% (v/v). Since these voxels are not connected to the roots or to each other they were not counted in the quantification of root length unless they were directly adjacent to the roots. This also points to a high water content at the bottom of the pot as the reason for failed CT root segmentation in this volume. However, we occluded roots found by MRI in this volume from root length quantification in order to compare similar volumes for both CT and MRI measurements.

For the left side of the medium pot there was no hint in the MRI image (Figure 2a) of high water content. The failure in root segmentation by CT in this region might be therefore different from what has been discussed before. Another problem in CT root segmentation can be an imprecise removal of the pot material that accidentally removed root segments from the image during the image processing pipeline. If part of a major root is removed, all the attached roots will also be lost for root length calculation if the resulting gap is too large. For MRI this was not an issue since PVC, similar to most other common plastic materials, gave no detectable signal at the applied measurement settings and therefore did not show up on the images. Nevertheless, the strong difference in root length extracted from the medium pot by CT or MRI (2.8 m and 4.6 m respectively), was unlikely based exclusively on this effect. With reference to the WinRHIZO data, MRI found 95% of the roots which, considering that the lowest part of the pot was not used for the MRI root length calculation, advises root length to be somewhat overestimated here by MRI. Possibly, some signal caused by high water content in the soil adjacent to the roots could not be excluded from root length quantification. Therefore, also for MRI it seems to be advisable to investigate effects of high soil water content, probably close to or above field capacity, for the specific plant-soil system on root detection and root length quantification as a basic control.

### For the large pots, MRI showed a larger fraction of the root system and more details compared to CT

**Figure 3:**
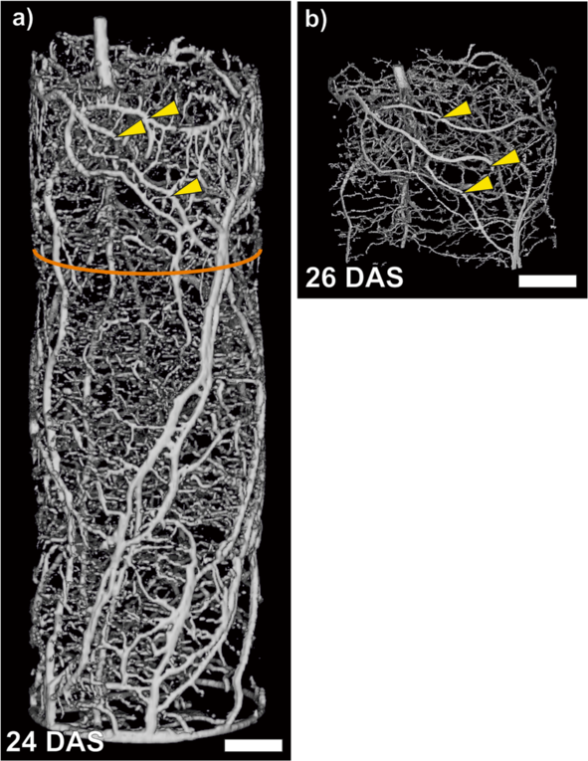
**MRI and CT images of a bean root system in a large pot.** The same plant grown in a soil filled pot with an I.D. of 81 mm and a height of 300 mm was imaged sequentially with MRI and CT. **(a)** MRI image shows the roots system in the whole pot with a voxel size of 521 × 521 × 1000 μm^3^. Root systems of the same plant segmented from CT data **(b)** on a voxel size of 99 × 99 × 99 μm^3^. Since the CT dataset was not complete only data of the upper ~75 mm of the pot could be reconstructed and are shown here. The orange colored ring denotes the volume measured also by CT. Arrowheads highlight the same roots in both images. Scale bar: 20 mm.

The biggest pots used here had an inner diameter of 81 mm and were 300 mm long. Again the roots system of a bean plant was consecutively imaged by MRI (Figure 3a) and CT (Figure 3b). A large number of the roots was found in the CT image with a range of diameters allowing root class identification. However, at this pot size, some roots appeared to have no laterals in CT while, on the same roots, they were visible in the MRI image. The MRI scan was accomplished two days prior to the CT scan, illustrated by one thick root which had elongated clearly (middle arrowhead), so growth of the laterals can be excluded. This indicates that, for larger pots, MRI may have a better imaging capability compared to CT. Also, the detected root length with MRI was about 50% higher than with CT (Table 1). A likely explanation for this is the loss in spatial resolution necessary to cope with larger pots. Segmenting roots with CT mainly relies on the high spatial (voxel) resolution achievable in a particular measurement, allowing extraction of root structures despite the low contrast between the roots and the soil background [3,19]. Due to the tradeoff between sample size and resolution the voxel size had to be adjusted to 99 μm for the large pot, compared to 56 μm for the small pot, where both methods performed equally. The resolution in MRI also had to be adjusted as well, from 333 μm to 521 μm, but since MRI root detection was based on the strong root-soil contrast allowing sub-voxel root detection it was much less affected by a loss in spatial resolution.

## Conclusions

Both CT and MRI produced high quality 3D images of root systems *in vivo* as demonstrated here for bean plants grown in soil-filled pots of three different sizes (34, 56 and 81 mm in diameter). For comparison, root lengths of the same plants were calculated from data obtained in consecutive measurement with both modalities. The obtained length of the investigated root systems and image details were distinctly different between CT and MRI and, in particular dependent on the pot size. Compared to CT, MRI was less affected by a lower spatial resolution associated with larger pot sizes and appeared to be better suited for imaging large root systems, e.g. at later developmental stages. On the other hand, CT gave almost identical results as MRI for small pots with respect to root length but provided more detailed information such as finely graduated root diameter estimation, in particular of small roots, due to higher spatial resolution. The possibility to visualize soil structures is a further intriguing feature of CT while MRI opens the possibility of measuring the water content of roots or soils. Considering the time needed to analyze a full root system, the scan time for both modalities was comparable but segmentation of roots from CT images took much longer than the scans while MRI data could be used for quantification immediately. Segmentation of the CT data, however, may be performed separately while scanning on other plants continues. According to WinRHIZO data, however a considerable portion of the root system present could not be detected by either MRI or CT even in the small pots. Most likely these are roots thinner than 400 μm. Altogether combining the two modalities on the same samples would be intriguing as, with direct root and water content detection by MRI and soil structure imaging by CT, many questions regarding root-soil interactions can be addressed. Interesting sub-volumes identified with MRI in larger pots could then be targeted and investigated in detail with high resolution CT. As demonstrated here, both methods have their specific pros and cons, but are excellent tools to non-destructively investigate properties of roots growing in soil.

## Methods

### Plant material

Common bean plants (*Phaseolus vulgaris* L. cv. ‘Shiny Fardenlosa’) were grown in a growth chamber from seed in a mixture of homogenized agricultural topsoil and coarse sand (1:9; v/v). A mix with less sand and the same soil was already shown to be suited for MRI application [35]. The agricultural soil, characterized as a gleyic cambisol, was collected by removing the top 30 cm from an agricultural field (Kaldenkirchen, Germany) and air dried. Subsequently, the soil was powdered and homogenized in a drum hoop mixer (J. Engelsmann, Ludwigshafen, Germany), sieved to 2 mm and freed of stronger ferromagnetic particles by moving it in a thin layer on a conveyor belt through a magnetic field provided by rare earth magnets (NdFeB N42, 1.3 T; Webcraft GmbH, Gottmardingen, Germany). Coarse quartz sand (grain size 0.71 to 1.4 mm; Quartzwerke Witterschlick, Alfter, Germany) was similarly freed of ferromagnetic particles. The ready mixture was filled into PVC tubes of three different sizes with (a) an inner diameter of 34 mm, a height of 200 mm, a volume of 0.182 l, (b) and I.D. of 56 mm, a height of 200 mm, a volume of 0.49 l or (c) an inner diameter (I.D.) of 81 mm, a height of 300 mm and a volume of 1.5 l. All had holes drilled into their bottom caps for drainage and aeration covered with nylon mesh (grid size 200 μm) to prevent loss of substrate and roots growing out.

The pots were watered to above container capacity and, after excess water had drained away, seeds were laid down in holes 2 cm deep and covered with soil. After germination the pots were watered automatically once per day with tap water. The growth chamber was set to 16 h light/ 8 h dark and 20°C/16°C, respectively, while relative humidity was kept constant at 60 ± 3%. Lighting was provided by 5 × 400 W HPI and 5 × 400 W SON-T lamps (both Philips, Hamburg, Germany) which alternated every 2 hours with 5 min overlap giving PAR intensity between 350 and 450 μmol m^−2^ s^−1^ at canopy level.

### Computed tomography

For the root detection we used the CT setup at the Fraunhofer Development Center X-ray Technology (EZRT) [23]. The CT setup was used with a FXE 225.99 X-ray tube and a Perkin Elmer XRD 1620 detector, which is a flat panel type and operates at a frame rate of 2 images s^−1^ in 14-bits full frame mode (2048 × 2048 pixels).

The measurement parameters were adapted to the three different pot sizes to ensure an optimal image quality. The tube was operated in ‘high power’ mode. Measurement parameters for the different pot sizes are listed in Table 2. For the 34 mm and 56 mm pots data set with and without a 1 mm Copper filter between X-ray tube and sample were acquired to investigate the effect of beam hardening on the image quality. Although the filter did remove the beam hardening artefact, this artefact was nevertheless only present on a thin surface layer of the PVC pot (Additional file 2). As the image quality inside the pot was identical for both settings, the used filter was found to be of no importance in this study. Since the pots were 200–300 mm long, multiple scans of the samples were made at different heights. The obtained single part volumes were merged together afterwards to a whole recording of the plant.

**Table 2:** **Parameters of CT measurements**

	**I.D. 34**	**I.D. 56**	**I.D. 81**
**Tube voltage [kV]**	220	220	160
**Tube current [μA]**	180	180	160
**Power [W]**	39.6	39.6	25.6
**Exposure time [ms]**	499	499	999
**Filter**	Cu 1 mm	Cu 1 mm	none
**Number of projections**	1200	1200	1200
**Voxel size [μm]**	28	34	49.5
**FOV in horizontal plane [mm]**	33	58	82
**FOV vertical [mm]**	74	104	75
**Measurement time [min]**	40	60	20*

*For the upper 75 mm that could be segmented as shown in Figure 3b. Total scan time for the whole pot ca. 1.5 h.

I.D. pot inner diameter [mm], FOV field of view, Voxel were isotropic with the same size in all three dimensions.

The scans were made with continuous sample rotation (fly-by) over 360°. For improved handling and decreased calculation times the root structure was analyzed after a 2 × 2 binning with a voxel sampling of 56 μm for pot size 34 mm, 68 μm for pot size 56 mm and 99 μm for pot size 81 mm. The effective dose to the sample during the scan was measured with an additional pot in the center of which a Dose Rate Meter 6150 AD with Scintillator Probe from automess (Automation und Messtechnik GmbH, Ladenburg, Germany) was buried in the same soil and subjected to the same X-ray exposure as the plant samples described above. This measurement gave a value of 274 mGy.

### Segmentation of roots from CT images

For the root segmentation we used the Volume Player Plus (VPP) software of the EZRT and the Modular Algorithms for Volume Images (MAVI) software package (Fraunhofer ITWM Kaiserslautern, Germany). In the first step the pot material was removed by manually fitting an ellipse to the inner pot wall in the first slice and removing everything outside this ellipse in all slices of the vertical stack. In cases where thin roots are directly abutting the pot wall or the pot is not perfectly rounded this may cause the loss of root voxels. Afterwards the images were binarised using a range of different gray values. First we determined for every data set which gray value area contains the roots. This area is quite wide due to the different thickness of the roots. Big roots show higher gray values than small ones. According to this we limited the gray value area by defining gray value ranges for small, middle and big roots based on empirical knowledge. In summary we cover the whole gray value area containing roots, but split this area in three parts linked to root thickness by hysteresis binarization. As a result we receive three partial volumes to work with. In the following steps every partial volume is cleaned from remaining non-root parts (described later on). At the end the three volumes containing small, medium and big roots were combined to one volume again and a closing algorithm is used to generate a closed architecture. The closing algorithm is dilation followed by erosion of the objects and was used with a closing radius of three times the respective voxel size. With the MAVI software the remaining non-root parts in the binarised image like stones, soil and air or water filled pores were removed using the labeling function and the object filter. The labeling function assigns to every connected object in the image a unique value. For defining the connectivity of the objects a neighborhood system is used. The information to the different labels is stored in the image history. With this history an object filter can separate them working with object size and sphericity. Small and roundish labels are defined by their sphericity as non-root objects and removed from the image. A sphere would have a value of 1 and a line one of 0. The threshold was set to 0.3. Further information about the image processing can be found in the textbook written by Ohser and Schladitz [45]. The whole procedure for segmenting roots took 1 h for one dataset, in case of the 34 mm pots 2 and for 56 mm pots 3 datasets were acquired. Both pots were of the same height but for the small pots MRI showed that they had not reached the bottom. In case of 81 mm pots 4 datasets were necessary to cover the whole pot, whereas only one could be used for segmentation.

### MRI measurement

MRI measurements were performed on a plant dedicated vertical bore 4.7 T magnet equipped with gradient coils providing up to 300 mT m^−1^ (Varian, Palo Alto, USA). For plants grown in 34 mm and 56 mm I.D. pots we used a 63 mm I.D. RF coil (sensitive vertical length 60 mm; Varian, Palo Alto, USA). For plants grown in 81 mm I.D. tubes we used a 100 mm I.D. RF coil (sensitive vertical length 100 mm; Varian, Palo Alto, USA). Experimental control was run on a Varian VNMRS console and a Linux PC using the Varian software VnmrJ. For 3D images a multislice spin echo sequence was used (single echo; provided as part of the instrument package by Varian). Measurement parameters are listed in Table 3. For the standard MRI data handling, several tools were written in IDL (ITT, Boulder, USA), including data re-ordering, Fourier transformation, filtering and concatenation of blocks of virtual slices that were generated for each pot. Three-dimensional image rendering was performed using MeVisLab (Mevis Medical Solutions AG, Bremen, Germany).

**Table 3:** **Parameters of MRI measurements**

	**I.D. 34**	**I.D. 56**	**I.D. 81**
**Voxel size in horizontal plane [μm]**	333	375	521
**Voxel size vertical [μm]**	1000	1000	1000
**Repetition time T**_**R**_ **[s]**	3	3	3
**Echo time T**_**E**_ **[ms]**	9	9	9
**Number of averages**	2	2	2
**FOV in horizontal plane [mm]**	64	72	100
**FOV vertical [mm]**	156	160	300
**Measurement time [min]**	40	40	60

I.D. inner pot diameter [mm], Voxel were anisotropic with the same size in the two horizontal dimensions and a larger size in the vertical dimension.

### Segmentation of roots from MRI images

Segmentation of roots from MRI images was achieved using a simple threshold on the MRI signal. The threshold was set to four times the noise level of the MRI data. For the medium sized pot, the excessive soil water was visible in the MRI images. For this data set the threshold was manually increased to remove the soil voxels.

### Image analysis

Prior to root length calculation, the MRI images were co-registered to the CT data by automated image registration using the program FSL [56] and interpolated onto the same grid. As some parts of the CT images could not be segmented, this co-registration step assures that the images of both modalities contain the same part of the root system. Furthermore, effects due to different voxel sizes are avoided by using the same data grid. Root length calculation was done as described by [57], with some adaptations to be able to process not only MRI images but also segmented root masks obtained from the CT data. First, to be able to process the data with our developed software tools the images were downsampled by an integer factor, resulting in isotropic voxel sizes for the individual plant between 300-400 μm. The root system architecture was then extracted by calculating the cheapest path [58] from each segmented root voxel to the manually selected shoot. In Schulz et al. [57], the cost of a path element was determined by the geometrical length and the MRI image intensity at the respective position. For the CT data, the actual image intensity is not a useful measure to describe the root structure, thus we only worked with the segmented root mask. As the ‘image value’ for the segmented mask was either 0 or 1, all paths had equal costs and multiple parallel traces in one root segment did occur during the root extraction. These were avoided by additionally weighting the path costs with the distance transform of the root mask, thus favoring paths that run in the center of the segmented mask. The resulting root trees then needed pruning: paths shorter than 3 mm were removed, as well as paths bridging more than 3.5 mm of non-segmented voxels. After visual inspection of the resulting root system architecture, the total length was finally calculated from it.

## Additional files

Additional file 1:
**Root length data for additional 34 mm pot measured with CT, MRI and WinRHIZO.** A second bean plant grown under the same conditions as the one shown in Figure 1 was also analyzed with CT, MRI and WinRHIZO. Here the results of the root length measurements for all three methods are shown. Additional file 2:
**Comparison of 34 mm pot scanned with CT with and without filter.** The same bean plant grown in soil in a 34 mm I.D. pot was scanned with CT a) with a 1 mm thick Copper filter on the X-ray tube at 220 kV tube voltage and 100 μA tube current and without the filter at 100 kV and 120 μA. The same horizontal slice from both measurements was selected and is shown here, depicting roots within the sand/soil matrix. The X-shaped structure is the cross section of a plastic support for the bean pushed into the soil. The white ring at the outer circumference of the pot in b) indicates beam hardening artefacts in this area.
